# Myalgic encephalomyelitis/chronic fatigue syndrome from current evidence to new diagnostic perspectives through skeletal muscle and metabolic disturbances

**DOI:** 10.1111/apha.14122

**Published:** 2024-03-14

**Authors:** Tiziana Pietrangelo, Stefano Cagnin, Danilo Bondi, Carmen Santangelo, Lorenzo Marramiero, Cristina Purcaro, Raphael Severino Bonadio, Ester Sara Di Filippo, Rosa Mancinelli, Stefania Fulle, Vittore Verratti, Xuanhong Cheng

**Affiliations:** 1 Department of Neuroscience, Imaging and Clinical Sciences, University “G. d’Annunzio” of Chieti-Pescara, Chieti, Italy; 2 IIM-Interuniversity Institute of Myology, University “G. d’Annunzio” of Chieti-Pescara, Chieti, Italy; 3 Department of Biology, University of Padua, Padova, Italy; 4 CIR-Myo Myology Center, University of Padua, Padova, Italy; 5 Department of Psychological, Health and Territorial Sciences, University “G. d’Annunzio” of Chieti-Pescara, Chieti, Italy; 6 Department of Bioengineering, Lehigh University, Bethlehem, Pennsylvania, USA; 7 Department of Materials Science and Engineering, Lehigh University, Bethlehem, Pennsylvania, USA

**Keywords:** differential diagnosis, exertional malaise, ME/CFS, pathophysiology

## Abstract

Myalgic encephalomyelitis/chronic fatigue syndrome (ME/CFS) is a demanding medical condition for patients and society. It has raised much more public awareness after the COVID-19 pandemic since ME/CFS and long-COVID patients share many clinical symptoms such as debilitating chronic fatigue. However, unlike long COVID, the etiopathology of ME/CFS remains a mystery despite several decades’ research. This review moves from pathophysiology of ME/CFS through the compelling evidence and most interesting hypotheses. It focuses on the pathophysiology of skeletal muscle by proposing the hypothesis that skeletal muscle tissue offers novel opportunities for diagnosis and treatment of this syndrome and that new evidence can help resolve the long-standing debate on terminology.

## 1 | ME/CFS: WHAT ARE WE DEALING WITH?

Myalgic encephalomyelitis/chronic fatigue syndrome (ME/CFS) is a debilitating illness constituting a health issue worldwide. Prevalence from a meta-analysis was reported to be 0.68% (95% CI: 0.48–0.97) globally in 2020; over a quarter of ME/CFS patients were reported to be house- or bed-bound, over half unemployed, and only 19% work full-time.^1^ A factsheet from the European ME Coalition (EMEC) pointed out that in 2020, approximately 2 million European citizens were suffering from the illness, with an annual burden of approximately 40 billion.^2^ The American Myalgic Encephalomyelitis and Chronic Fatigue Syndrome Society reported that more than 1 million people in USA suffer from ME/CFS with an economic impact in the range of 36–51 billion dollars per year.^3^ Worldwide, the number of people affected by the condition may be as many as 17–24 million. Despite decades of research, definitive tests or biomarkers for objective diagnosis have yet to be established. The severe case of ME/CFS is further aggravated by the large number of long COVID patients, who share symptoms with those with ME/CFS, highlighting the public health impact of chronic fatigue following an infectious episode. Thus, there is an urgent need to accelerate research on ME/CFS for both diagnostic and therapeutic purposes.^4^

ME/CFS is a peculiar disease with debilitating symptoms such as intolerance to exercise and abnormal fatigue and malaise after physical or cognitive efforts at intensities previously tolerable to individuals. The marked and prolonged exacerbation of symptoms, termed post-exertional malaise (PEM), may last several days. Furthermore, pain, sleep disturbances and anxiety/depression are typically present as debilitating clinical features, according to the diagnostic criteria described in the well-accepted criteria.^5–9^ In 2015, the Committee on the Diagnostic Criteria for Myalgic Encephalomyelitis/Chronic Fatigue Syndrome (Institute of Medicine, National Academies, USA) proposed the name systemic exertion intolerance disease (SEID) to link the main pathognomonic sign to this disease.^10^ That proposal was aimed to solve the disagreement as to whether ME and CFS are similar enough to belong under the umbrella term ME/CFS; the proposed diagnostic criteria of SEID focused on unexplained fatigue, functional impairment, and PEM, whereas muscle-related symptoms disappeared. Skeletal muscles of patients show peculiarities nevertheless.

## 2 | FATIGUE AND MUSCLE FATIGUE IN ME/CFS

ME/CFS is marked by both central and peripheral fatigue. Central fatigue is represented by decreased recruitment of motor neurons which leads to highly fatiguing motor units. Peripheral fatigue, on the other hand, is caused by muscle fibres’ inability to contract as a result of several altered metabolic processes, such as oxygen imbalance and modified flux of calcium or potassium. In patients both conditions can be measured: central fatigue is assessed by measuring the restoration of a contractile response during fatiguing attempts, while peripheral fatigue can be observed when the contractile response under direct electrical muscle stimulation is reduced.^11^ Both conditions finally cause muscle fatigue (MF), a condition characterized by decreased power production and incapability to generate the necessary amount of force to maintain muscle contraction.^12,13^ When MF is caused by accumulation of byproducts of intermediary energy metabolism or depletion of energy-rich compounds related to demanding physical activity, it is defined as temporary muscle fatigue. Otherwise, when MF is caused by muscle atrophy, which also occurs due to sarcopenia, muscle disuse or neurogenic impairment, it is defined as chronic muscle fatigue.^14^

MF is one of the main symptoms of ME/CFS reported by patients after moderate physical activity. In fact, difficulty in maintaining muscle activity is broadly described and it seems to be caused by lack of energy or muscle pain, with the latter underlying the neurological involvement.^15^ In order to evaluate muscular strength and to obtain information regarding patient’s state of health and physical function, measurement of hand grip strength (HGS) and recovery time from fatiguing exercise are used. Several studies highlighted the decrease in HGS and impaired recovery in people affected by ME/CFS, suggesting the clinical relevance of the HGS evaluation in this illness.^16–20^ A reduction in muscular strength has also been observed in the legs, where measurements of quadriceps force-matched HGS scores^16,21^; however, another study did not show muscle force reduction.^22^ With regard to the impairment of recovery, the work rate of ME/CFS patients at anaerobic threshold is reduced in the second out of two VO_2_ peak tests separated by 24 h compared to healthy controls, with a minimal clinically important difference of 10 W.^23^ Ample evidence shows that ME/CFS patients have reduced functional capacity in repeated cardiopulmonary exercise tests compared with healthy controls,^24–26^ supporting systemic exertion intolerance as a cornerstone of ME/CFS.

Moving from the paradigm of the failure of skeletal muscle contractile properties 20 years ago, fatigue has been identified as the general body alarm to preserve the physiological homeostasis and integrity of the body. The dichotomy between central and peripheral fatigue has been unified by means of the more recent theoretical models of fatigue as cross-talk between central nervous system and muscle-dependent signals produced during exhaustive exercise.^27,28^ In this light, the fatigue in ME/CFS may reveal an altered cross-talk that originates from skeletal muscle that propagates into the central nervous system, since signals are integrated in the brain in order to stop physical exertion in the absence of exhaustive exercise. This hypothesis poses skeletal muscle as the central piece in ME/CFS. Skeletal muscle has systemic effects regulating, for example, immunological processes and inflammatory response.^29^ Systemic inflammation, vice versa, is associated with skeletal muscle atrophy and metabolic dysfunction^30^ in conditions such as the chronic obstructive pulmonary disease.^31^ Similar impacts of the skeletal muscle on other systems can occur in ME/CFS, leading to the observed multi-system symptoms. In fact, skeletal muscle has been extensively studied, from mechanistic hypotheses of pathogenesis and emergence of symptoms to novel strategies for the treatment.^15,32,33^ The current work aims to provide an overview of recent knowledge on ME/CFS centered around, but not limited to, skeletal muscle, by reviewing the compelling evidence and most interesting hypotheses. In this work, although the debate on whether or not ME and CFS should be distinguished entities, we will use the umbrella term ME/CFS, as used by groups and societies (e.g., “EUROMENE” and “American ME and CFS Society”) and in most studies.

## 3 | PATHOPHYSIOLOGICAL MECHANISMS

The musculoskeletal disorders in ME/CFS patients at young ages have been described with the aphorism “old muscle in young body,” since the underlying physiological compromise mimics in some respects what happens during aging, especially fatigue.^34^ The next sessions will be focused on recent discoveries of ME/CFS pathophysiology in skeletal muscles.

### 3.1 | Mitochondrial and calcium homeostasis in fatigue and ME/CFS

A systematic review of 19 studies investigating mitochondrial structural and functional differences in ME/CFS patients compared with healthy controls has been published recently, aiming to elucidate mitochondrial abnormalities in ME/CFS. It suggests that ME/CFS is not a primary mitochondrial disorder despite consistent genomic results. Instead, mitochondrial decline might occur due to secondary effects of other disrupted pathways.^35^ Nonetheless, mitochondria likely assume a pivotal role in ME/CFS pathophysiology. A recent study of the bioenergetics of peripheral blood mononuclear cells reveals that abnormalities in mitochondrial functions occur in all ME/CFS patients regardless of the severity of the symptoms. However, higher cytosolic glycolytic acidification and lower mitochondrial ATP-dependent respiration rate have been correlated with the severity of the syndrome in peripheral blood-mononucleated cells.^36^

A recent paper hypothesized the role of the ß2-adrenergic receptor dysfunction in the pathophysiology of ME/CFS in skeletal muscle, thus affecting mitochondrial function in myocytes. In this paper, the authors hypothesized that ß2AdR dysfunction could cause sympathetic overactivity in both brain and skeletal muscle. Considering that ß2AdR in skeletal muscle normally stimulates the Na^+^/K^+^-ATPase pump, ß2AdR impairment leads to reduced pump activity, causing sodium overload that in turn activates both the sodium–calcium exchanger to operate in its reverse mode, importing calcium instead of exporting it, and decreases sodium–proton exchanger activity that acidifies myocyte cytosol.^32^ It is well established that cytosolic ion perturbation, such as cytosolic calcium, increases at the resting condition, and negatively impacts mitochondrial transmembrane hyperpolarization and organelle functions. Both cytosolic acidification and sodium-dependent calcium overload reduce the ATP rate. Since myocyte ATP demand sustains pump activities (up to 10%–25% of ATP for sarcoplasmic reticulum calcium-ATPase and 5%–10% of ATP for sodium–potassium exchange activity) and actomyosin ATPase activity (65%–80% of ATP),^37^ an insufficient ATP rate in ME/CFS muscles affects contraction features and exacerbates transport inefficiency, establishing fatigue condition that can assume the features of chronic fatigue syndrome. The cytosolic ion perturbation and insufficient ATP rate are exacerbated when skeletal muscle engages in sustained excitation–contraction cycles during exercise. Furthermore, the cytosolic calcium overload negatively impacts the sarcoplasmic reticulum storages and their calcium-binding protein activities for calcium release and uptake. Similarly, the altered resting calcium level or protein activity deficiency might establish oxidative damage by increased reactive oxygen species (ROS) production due to real mitochondria rupture or indirect mitochondria reaction (Figure 1). There is also evidence for an inhibition of the Na^+^/K^+^-ATPase by ROS in ME/CFS patients that may stem from inflammatory processes or mitochondrial dysfunction, exacerbating the previously mentioned metabolic disruption.^32^

In our previous work,^55^ weaker nicotine-dependent Ca^2+^ mobilization was observed from ME/CFS myotubes, likely due to reduced acetylcholine-dependent channels in the plasma membrane and/or less efficient nicotine-stimulated opening. We expect that this cellular characteristic is present in ME/CFS and absent in fibromyalgia, given the differences in phosphocholine expression. This differential aspect could be directly linked to defective acetylcholine formation and is typical of ME/CFS.

### 3.2 | Oxidative stress in fatigue and ME/CFS

It is widely accepted that while regular exercises have health benefits, exacerbated physical activities result in an increase in the production of reactive oxygen species (ROS), thus causing oxidative stress.^38^ In the past few decades, oxidative stress was considered an imbalance between oxidant and antioxidant species in favor of the former.^39^ Living cells, particularly myocytes, produce many types of free radicals counteracted by enzymatic and non-enzymatic antioxidant systems.^40^ Early studies conducted in 1990s suggesting the correlation between ROS and skeletal muscle fatigue stimulated research to elucidate the underpinning mechanisms. Several antioxidant enzymes have been found to overproduce following exercise training, such as superoxide dismutase (SOD) isoforms, glutathione peroxidase (GPX1), and catalase (CAT).^38^ Further studies clarified that ROS are signalling molecules to activate the expression of specific genes expression in muscle and other tissues.^41^ Indeed, skeletal muscle is considered the main source of oxidants during exercise,^42^ mainly produced by mitochondria,^43^ along with phospholipase A2^44^ and nitric oxide synthase.^45^ The evidence collected in early 2000s demonstrated that ROS has a biphasic effect on skeletal muscle, revealing that an ideal amount of ROS is essential for the generation of maximum force in muscle fibres, while an excessive ROS production decreases muscle force.^46^ The possible mechanisms responsible for the ROS impact on muscle force production are still debated; however, some research suggests that over a certain threshold, ROS reduces both the myofibrillar calcium sensitivity and consequently the muscle force production.^47^ Moreover, as mentioned before, an excessive ROS concentration inhibits the Na^+^/K^+^ pump activity that in turn establishes muscle force reduction.^48^ From another point of view, some increase in the amount of ROS produced during exercise (a physiological package of ROS) is critical for muscle adaptation to exercise because it mediates the activation of major skeletal muscle metabolic signalling such as the NF-kB, PGC-1α, and mTOR pathways.^49,50^ In this vein of evidence, when excessive ROS could be considered dangerous, one way to regulate the oxidant levels in the cells is to use integrators or drugs that reduce ROS concentration or activate the antioxidant system, as proposed by our and other groups.^51^ Overall, the role of redox balance in the occurrence of fatigue has been largely debated. On the one hand, it seems that prolonged oxidative stress due to exercise in muscle is unlikely, an assumption supported by exercise-induced hormesis. Thus, an active lifestyle is recommended to maintain a healthy status.^52^ On the other hand, oxidative stress is considered a potential risk factor or enhancer of many pathological conditions. The discrepancy could come from the persistence and accumulation of ROS in working muscle. ROS accumulation contributes to the loss of function which represents a proxy of fatigue. Following this argument, an efficient strategy could be to establish an antioxidant therapy and monitor those who show premature fatigue to see eventual benefits.^53^

Some oxidative molecular damages have been found in skeletal muscle of ME/CFS, specifically increased membrane rigidity and cytosolic [Ca^2+^]i elevation and decreased antioxidant enzyme activity.^54^ Fluidity and fatty acid composition in ME/CFS muscle membranes are significantly different from those of the controls.^54^ The typical symptoms of muscle weakness and fatigability in the presence of non-atrophic contractile muscle suggest a very complicated framework in which skeletal muscle is able to reveal the biological origin of the syndrome. It could be argued that impairment of the redox network is a common feature in many physio-pathological conditions such as aging. However, substantial difference exists in the biological machinery involved in the two conditions. Skeletal muscle of ME/CFS patients shows no sign of atrophy along with significant downregulation of FOXO, the master gene involved in atrophy,^55^ while elderly show atrophic/sarcopenic muscles with FOXO activated, despite a wide range of heterogeneity.^56^ The altered signalling, directly or indirectly, takes part in the weakness and fatigability manifestation in the muscle of ME/CFS, but more importantly, all of them directly or indirectly depend on the muscle’s physiological homeostasis and metabolism.

The physiological consequences of a shortage of ATP-bearing energy and excessive lactate production are in agreement with the debilitating exercise intolerance found in patients with ME/CFS.^57^ This intolerance could be explained also by our results that support the hypothesis of an increase in oxidative stress in the ME/CFS skeletal muscles, mainly dependent on increased production of free radicals instead of a decreased efficiency in the antioxidant system. Specifically, the main source of free radicals’ overproduction is linked to maximization of mitochondrial oxidative phosphorylation in response to ATP demanding, amplified during exercise. The pictures of ME/CFS mitochondria captured by electron microscopy show poly- and pleiomorphism with thickening of mitochondrial cristae.^54^ The altered mitochondria morphology in human myotube, along with significantly increased cytosolic glycolytic rate, show similarities between elderly and ME/CFS young subjects^58^ (Figure 1).

The signs of oxidative stress and fatigability, as similarly documented in aging, argue the aphorism “old muscle in young body” for ME/CFS, since the underlying physiological compromise, at the musculoskeletal level in ME/CFS, imitates to some extend what happens while aging.^34^ Interestingly, the features in aged skeletal muscle are also represented by aged human muscle precursor cells, both sharing the characteristics of increased membrane rigidity, cytosolic [Ca^2+^]i elevation, decreased antioxidant enzyme activity, and less efficient differentiation.^59,60^ In the same vein, the adaptability of muscle precursor cells to chronic ROS stressors opens the way to future studies of adult myogenesis to reveal the peculiar microenvironment in ME/CFS, including possible autocrine and paracrine signals from immune cells and adipogenic and fibrogenic progenitors.

### 3.3 | Satellite cells: Possible roles in ME/CFS

At the base of muscle homeostasis, there is the regenerative process, the ability of skeletal muscle to rebuild itself by means of cell turnover, as in the case of tissue damage or injury. This specific muscle plasticity depends on the human satellite cells (SCs), adult stem cells residing beneath the basal lamina over the plasma membrane of skeletal muscle fibres.^61^ This stamina pool intervenes continuously in muscle repair during human life and reflects genetic, epigenetic, and environmental adaptation of fibres, a feature very precious making skeletal muscle a plastic tissue that responds to insults, external factors, or genetic issues.^62–65^ Studies of SCs and stem cell-based approaches have found utility in treating genetic muscle diseases like dystrophy by replenishing the muscle stem cell pool.^66^

Compared to rich knowledge of oxidative stress (OS) in skeletal muscle, OS associated with ME/CFS in SC has not been deeply investigated. SCs may be influenced by their surrounding environment including soluble factors in the bloodstream, and adapt themselves to what adult fibres have gone through. In this vein, a simple mechanical short fibre unload has been found to modify the SC pool, by their premature differentiation via Wnt pathway activation.^67,68^ Therefore, there is a continuous cross-talk between SCs and myofibres and vice versa. Since clinical symptoms suggest that skeletal muscle is a major target of ME/CFS syndrome, and skeletal muscle precursor cells (MPCs) play a critical role in skeletal muscle repair and function, we support the hypothesis that SCs from the skeletal muscle tissue of ME/CFS patients are distinguishable from those of healthy individuals on the molecular and cellular level.

Currently, MPC-based strategies have been pursued in clinical practice for treating muscle loss using satellite cells, mesangioblasts, pericytes, and mesenchymal stem cells that show extensive proliferative capabilities when transplanted, overall, in scaffold-oriented regeneration.^69–71^ MPCs, used for reconstructing skeletal muscle tissue loss, functionally integrate themselves into the existing musculature of the host, showing a sort of scaffold-dependent success in regeneration. Biopsies of calf muscle showed growing myoblasts cells and muscular tubes and an improvement in arms and legs during physical examination was reported. Moreover, MPCs have been involved in myotube formation through heterotypic cell fusion after myogenic gene activation.^69^

Stem-cell-based therapies provide notable therapeutic benefits in reversing muscle atrophy and promoting muscle regeneration, showing the best positive results for treating Duchenne muscular dystrophy.^72–74^ However, a significant limitation exists for the therapeutical usage of MPCs due to the significant reduction in their ability to proliferate and consequently, obtaining a sufficiently large number of fresh SCs for clinical application when a large amount of muscle has to be rebuilt.^75,76^

### 3.4 | Epigenetic aspects in fatigue and ME/CFS

As previously described, one of the primary symptoms in CFS patients is muscle fatigue, but biological basis of the pathology remains poorly understood. However, recent research has suggested that epigenetic modifications, specifically DNA methylation, may be associated with CFS.^77^ Epigenetics is the study of heritable changes in gene expression or cellular phenotype that do not involve alterations to the DNA sequence. It is already known that epigenetic modifications play a crucial role in a wide range of biological processes, such as embryonic development,^78^ cell differentiation,^79^ aging,^80^ and disease susceptibility,^81^ and can be influenced by various factors, including environmental exposures such as diet, stress, or toxins^82^ and lifestyle choices such as exercise and sleep pattern.^83^ The main types of epigenetic modifications include:

- DNA methylation, which typically leads to gene silencing, prevents the binding of transcription factors and other regulatory proteins to the DNA, thereby reducing gene expression.
- Histone modifications by acetylation (associated with active gene transcription), methylation (which can either activate or repress gene expression), phosphorylation, and ubiquitination.
- Non-coding RNAs, such as microRNAs (miRNAs, which bind to mRNAs), long non-coding RNAs (lncRNAs, which can act as scaffolds for chromatin-modifying enzymes), and circular RNAs (circRNAs, which usually interact with miRNAs and act as competitive endogenous RNAs, “sponging” miRNAs, and modulating biological pathways) (Figure 2).

Understanding epigenetic mechanisms is crucial for unraveling the complexities of gene regulation and disease development. It offers potential insights into the disease risk, prognosis, and therapeutic intervention by targeting enzymes regulating epigenetic alterations to restore normal gene expression patterns and cellular functions.

Several studies have suggested that epigenetic modifications may contribute to the development and maintenance of fatigue and ME/CFS.^77,84–93^ DNA methylation was studied in individuals with ME/CFS permitting the identification of epigenetic alterations, particularly in genes related to immune system regulation, energy metabolism, and stress response. These changes may disrupt normal cellular processes and contribute to the symptoms observed in ME/CFS, including fatigue. An interesting concept that recently was proposed is the dynamicity of epigenetic alterations associated with the dynamicity of ME/CFS.

The epigenetic landscape of ME/CFS was evaluated by several research groups. The DNA methylation analysis of CD4+ T cells revealed 120 CpG islands differentially methylated in the CFS/ME patients compared to the healthy donors, most of them hypomethylated.^88^ Those alterations were associated with genes that participate in apoptosis, cell development, cell function, and metabolic activity processes. The differential methylation analysis of De Vega and collaborators identified a global hypermethylation in the peripheral blood mononuclear cells (PBMCs) of ME/CFS, but a specific hypomethylation within promoters and gene regulatory elements of genes involved in immune response, cellular metabolism, and kinase activity.^77^ Interestingly, DNA hypomethylation associates ME/CFS to aging where an overall decrease in global DNA methylation occurs resulting in the relaxation of gene expression regulation and abnormal gene expression.^94^ A study from De Vega and colleagues with a larger cohort of female patients revealed 13 loci differentially methylated associated with glucocorticoid sensitivity.^95^ This finding could explain the hypothalamic–pituitary–adrenal (HPA) axis dysfunction in ME/CFS. In support of previously described results, Vangeel and collaborators identified a DNA hypomethylation of the glucocorticoid receptor gene NR3C1 in the blood samples from female CFS patients.^96^ The authors state that this DNA methylation pattern could be a consequence of early life stress exposure or childhood trauma, a possible etiology factor for ME/CFS, since the glucocorticoid receptor plays a key role in the hypothalamic–pituitary–adrenal (HPA) axis response by exerting a negative feedback mechanism. However, due to the cross-sectional nature of the study, the causality effect remains elusive.

The association of genetic and epigenetic features was assessed by Herrera and collaborators.^85^ They detected aberrant methylation in close proximity to genes involved with immune function and cellular metabolism, but no direct genotype–phenotype associations. However, the differentially methylated loci had significant correlations with specific genotypes at neighboring SNPs, suggesting that particular genetic backgrounds may influence methylation levels in ME/CFS patients.

Trivedi and collaborators used a microarray-based approach to identify 17 296 differentially methylated CpG sites in 6368 genes across regulatory elements and within coding regions of genes, most of them related to cell signalling and immune regulation.^86^ Helliwell and colleagues by analyzing DNA methylation suggested that ME/CFS is associated with dynamic epigenetic changes, and these changes appear to be influenced by the different phases of the condition.^90^ Systemic dysfunctions associated with ME/CFS were linked to alterations in DNA methylation patterns and immune, metabolic, and neurological pathways.^88^

As previously stated not only DNA methylation is responsible for epigenetic alterations but histone modifications are also associated with the regulation of transcriptional program because their modifications, such as acetylation or methylation, can influence the accessibility of genes to the cellular machinery responsible for the gene expression. Altered histone modifications have been reported in individuals with ME/CFS, indicating potential dysregulation in gene expression that may contribute to the pathophysiology of the condition. For example, McGregor and colleagues recently proposed that post-exertional malaise (PEM), the cardinal predictive symptom in the definition of ME/CFS, may be a result of hypoacetylation and metabolite loss during the acute PEM response.^9^

Non-coding RNA molecules, including microRNAs, have also been implicated in ME/CFS and fatigue. These molecules do not code for proteins but can regulate gene expression by binding to messenger RNAs (mRNAs) and influencing their stability or translation into proteins. Studies have identified dysregulated expression of specific microRNAs in ME/CFS patients, suggesting their potential involvement in the disease process and fatigue.

To our knowledge, 11 studies reported alterations in miRNA expression in ME/CFS patients and those findings are summarized in Table S1. miR-127-3p and miR-142-5p were upregulated in plasma of ME/CFS patients.^91,97,98^ MiR-150-5p was reported in four studies, demonstrating that this miRNA plays an important role in the disease, but some results are contradictory probably due to the complexity of the disease. Cheema and colleagues detected higher levels of miR-150-5p in the PBMC fraction of ME/CFS patients.^99^ Nepotchatykh et al.^97^ and Soffritti et al.^98^ had similar findings in plasma samples, but Al-Rawaf and colleagues^100^ reported opposite results. MiR-146a-5p was downregulated in plasma,^100^ PBMC fraction,^99^ and natural killer cells^101^ of ME/CFS patients. Qin and colleague demonstrated the importance of miR-146a-5p in the communication of skeletal muscle with other tissues through exosomes.^102^ Its down-modulation in the plasma of ME/CFS patients may support the inability of skeletal muscle to correctly communicate with other tissues, which causes systemic alterations. According to two studies, miR-233 is downregulated in leukocytes from patients.^99,101^ MiR-92a and −93 were also reported by more than one study, but opposite results were reported as well. Baraniuk et al. detected a downregulation of these miRNAs in cerebrospinal fluid of ME/CFS patients after exercise,^103^ while Narayan et al. detected an upregulation of the same miRNAs using a similar experimental design.^104^ More studies should reveal the usefulness of miR-92a and −93 in biological applications. Blauensteiner et al., Almenar-Pérez et al., and Petty et al. provided a list of differentially expressed miRNAs associated with ME/CFS, but more validations are required since results are discordant with other papers.^105–107^ In summary, biological pathways related to altered miRNAs are mainly related to the regulation of immune response, inflammation, and cell cycle regulation (Table S1). Altogether, these studies might provide valuable insights into a molecular signature associated with ME/CFS.

Yang and collaborators examined the expression of 10 lncRNAs in the peripheral blood mononuclear cells of 44 ME/CFS patients and identified increased levels of NTT, MIAT, and EmX2OS in the patients.^108^ Moreover, the authors showed that NTT and EMX2OS expression levels highly correlated with ME/CFS disease severity. Both lncRNAs are involved in inflammatory response,^109–111^ but their precise role in ME/CFS is still unclear and further experiments might reveal the mechanism of action.

An RNA-seq analysis revealed a unique circRNA signature in ME/CFS patients.^112^ Fourteen circRNAs were reported to be upregulated in whole blood samples from ME/CFS patients: circDLEU2, circVPS13A, circFAM193A, circOGFOD3, circNPAT, circNCOR2, circASH1L, circPAPOLA, circFGGY, circPCNX2, circCSF3R, and circLIN54; and two isoforms of circNUP98. These RNAs are mostly involved with energy metabolism, oxidative stress response, and inflammation. Interestingly, the gene ontology analysis showed that three of those RNAs (circCSF3R, circLIN54, and circNUP98) could participate in ubiquitin-dependent protein catabolic processes, and their abnormal expression could participate in the exacerbated fatigue and post-exertional malaise typical in ME/CFS patients.

It is important to note that the field of epigenetics and its relationship with fatigue and ME/CFS is still evolving, and more research is needed to fully understand the complex interactions and mechanisms. Nonetheless, the emerging evidence indicates that epigenetic modifications have the potential to contribute to the development and persistence of fatigue and ME/CFS by influencing cell metabolism, inflammation, and glucocorticoid receptor activity. No one study considered skeletal muscle which is the most extended tissue that regulates whole body metabolism.^113–115^ and whose functionality is associated with inflammation. Interestingly, skeletal muscle mass may be hormonally regulated and glucocorticoids elicit the atrophy of muscle by increasing the rate of protein degradation through the ubiquitin–proteasome system and autophagy–lysosome system.^116^ Usually, muscle atrophy and inflammation are two leading causes underpinning the development of chronic fatigue.

### 3.5 | Genetic aspects in fatigue and ME/CFS

Epigenetics is the first step of gene regulation, while alterations in the DNA sequence may modify gene expression and influence phenotypes (Figure 2). It is known that alterations in 5-hydroxytryptamine (5-HT) signalling can lead to physiological and behavioral changes which also occur in ME/CFS. Smith and colleagues evaluated the 5-HT system genetically in order to establish serotonergic markers associated with ME/CFS; this study explored the influence of 77 SNPs in 14 genes involved in serotonin synthesis, signalling, metabolism, or transport.^117^ Interestingly, among examined polymorphisms, three results associated with ME/CFS (−1438G/A, C102T, and rs1923884). All polymorphisms are located in the 5-HT receptor subtype HTR2A. In another study by Carlo-Stella and colleagues,^118^ it was considered cytokine gene polymorphisms to identify a genetic predispositions to inflammation development. They discovered that the increased TNF level is a reliable marker for ME/CFS, which depends upon a genetic susceptibility due to the increased frequency of the TNF-857T allele. More specifically, the IFN-γ 874 AA genotype is associated with a low production of this cytokine. The IFN-γ AA genotype is less frequent in patients with ME/CFS than in controls. Therefore, ME/CFS patients manifest a proinflammatory profile that affects the immune response to environmental stresses and may explain the chronicity of the disease.

Since ME/CFS is a disabling condition characterized by unexplained chronic fatigue that impairs normal activities, it is important to establish which pathways might be involved in the onset and development of muscle symptoms. In a study involving 10 patients (5 women and 5 men), a global transcriptomic analysis was performed to identify genes that are differentially expressed in skeletal muscle (*vastus lateralis*) of ME/CFS patients in comparison with healthy subjects.^55^ The authors found altered expression of genes involved in the regulation of mitochondrial function, redox balance, energy production, muscular trophism, and fibre phenotype determination. Furthermore, it was established that the expression of a gene encoding a component of the nicotinic cholinergic receptor binding site was reduced in ME/CFS patients, indicating a possible impairment in the neuromuscular transmission. In a separate study by Li and colleagues, cultured human skeletal muscle cells were treated with serum derived from health and subhealthy subjects with fatigue. Genes alterations were associated with the cell cycle, membrane channels, protein transport, energy metabolism, and apoptosis,^119^ suggesting that fatigue may be communicated by circulating molecules. Although this study did not specifically focus on CFS, the insights into the gene expression changes associated with fatigue may be relevant to ME/CFS.

Skeletal muscle may be the target of secreted molecules that induce premature fatigue by altering muscular trophism and impairing neuromuscular communication.^55^ Therefore, investigations need to focus not only on the influence of a single aspect such as single nucleotide polymorphisms (SNPs) or differential gene expression on a tissue but also on the pathology with a systems biology approach that also considers cell communication. Recently, transcriptome analysis at the single cell level was performed on cells of the immune system,^120^ which allows the comprehension of intercellular communications. The authors evidenced improper platelet activation in patients may be associated with muscle fatigue. In fact, platelet-derived signalling factors are instrumental in guiding tissue regeneration, a process activated in the skeletal muscle after exercise.^121^

### 3.6 | The link between exposome and internal pathways

The biological interferences of environmental, psychological, and physiological stressors all integrate into the system linked to the gut–brain axis. Patients affected by ME/CFS commonly report sustained stress, infections, exposure to toxic agents, and traumatic events that may lead to epigenetic changes with a lasting impact on the immune and neuroendocrine systems. There are also evidences that highlight the synergy between alterations in the gut microbiota, mucosal barriers dysfunction, and immune response,^122,123^ but the pathological mechanisms are not yet established. An interplay between guts and muscles has been proposed. In fact, the gut–muscle axis seems to play an important role in muscle homeostasis considering the influence of the gut microbiota upon muscle mass loss and reduced muscle function in the aging process.^124,125^ Skeletal muscle cells can be influenced by several compounds produced or modified by the gut microbiota whose products act as nutrients or physiological modulators involved in muscle anabolism and prevention of muscle function loss.^126^ For example, folate, vitamin B12, short-chain fatty acids (SCFAs), and urolithins produced by the host microbiota are involved in (1) preservation of skeletal muscle cells through improvement of mitochondrial biogenesis and activity, and consequently energy production, (2) improvement of redox reactions, and resistance to fatigue, (3) promotion of muscle anabolism, and (4) activation of pathways that involving IGF-1 leads to promotion of genetic regulation of myofibrillar synthesis.^126^ A persistent shift in the microbial structure and diversity can lead to dysbiosis and pathological conditions such as metabolic disorders,^127^ autoimmune conditions,^128,129^ and neurological disorders,^130,131^ acting on DNA, inducing epigenetic changes^132^ and modulating host’s immune response.^133^ Several studies highlighted gut dysbiosis and an increased gut permeability in ME/CFS patients,^134–136^ but a clear or direct correlation is still missing (Figure 3).

There is evidence supporting bidirectional communication between gut microbiota metabolites and extra-intestinal organs, including the central nervous system (CNS) and peripheral excitable tissues. This communication is thought to be mediated by the translocation of gut metabolites through the circulatory system, triggering tissue-specific local immune responses^137^ and involving endocrine, neural, and metabolic pathways.^138^ Since the early stage of life, the gut microbiota can produce and interact with the brain through various mechanisms and mediators including cytokines, short-chain fatty acids, hormones, and neurotransmitters that affect development and function of the HPA axis.^139^ Stressful events experienced during life can influence both the microbiota composition and HPA axis, and dysbiosis can impact the development of the latter. In general, HPA axis activation with the consequent hormones cascade, ending in cortisol production, is a normal adaptation mechanism to environmental stressors.^140^ An abnormal activation occurs due to the downregulation of stress response through an increased negative feedback mechanism, with implications on immune–inflammatory pathways through bidirectional mechanisms.^141^

For what concerns immune regulation, a lack of regulation of the activity of the immune system in ME/CFS patients is specifically associated with abnormalities of immune cell phenotypes such as modification of the NKP46 SLAM receptor expression on natural killer (NK).^142,143^ NK cells dysfunction could be modulated by gut microbiome through bile acid modification and consequent impact on liver cell gene expression, leading to NK cell accumulation.^144,145^ This is only one of the immunological abnormalities found in ME/CFS, and alterations that are common in other immune or autoimmune diseases such as fibromyalgia, Hashimoto’s thyroiditis, and family history of autoimmune diseases have also been linked to ME/CFS.^146^ In particular, there is a relationship between the immune system and muscle. Indeed, reduction of the skeletal muscle fibre diameter and protein loss are caused by increased production of cytokine related to aging, including C-reactive protein (CRP), tumor necrosis factor-alpha (TNF-α), TNF-like weak inducer of apoptosis (TWEAK), ROS, and IFN-c. Myocyte apoptosis and muscle proteolysis can occur due to skeletal muscle metabolic malfunction.^147^ In this context, several studies highlighted cytokine pattern modification in ME/CFS. Moreover, the cytokine profile and immune cells seem to change across disease stages, however, their role needs to be further investigated.^148^ ME/CFS is also characterized by autoimmune processes. High levels of autoantibodies against ß2 adrenergic (ß2AdR) receptors and M3 acetylcholine receptors have been detected in ME/CFS patients. Line Sommerfeldt and colleagues demonstrated the association between polymorphisms of ß2AdR gene, the Gln27 mutation, and ME/CFS.^149,150^ Furthermore, ß2AdR is also connected with skeletal muscle because it takes part in muscle vasodilatation.^150^

### 3.7 | The metabolic disruptors hypothesis

Biological non-linearity results in multiple different steady states, depending on external conditions; organisms in a disrupted steady state are more vulnerable to perturbations that precipitate a shift to pathophysiology mechanisms and they would hardly reverse this shift. The “metabolic trap” hypothesis (see the Section 5.1) is based on this theory.^151^ Several studies postulated viruses as external perturbators that pathologically dysregulate metabolic functions, ultimately leading to chronic fatigue. Within this framework, in addition to infectious agents, biomolecule triggers represented by disrupting chemicals can also interfere with the metabolic steady state. Therefore, it would be interesting to evoke metabolism-disrupting chemicals as a class of endocrine disruptors which affect energy homeostasis and favor multisystemic disorders.^152^

The bioenergetic disturbance in ME/CFS remains enigmatic. A number of studies have indicated that the skeletal muscle tissues of ME/CFS patients show significant metabolic alterations involving energy production, muscular trophism, neuromuscular transmission, and fibre phenotype determination. Lactate overproduction and marked increase of intracellular pH after moderate exercise along with a lower rate of ATP synthesis during recovery^153^ and increased proportion of fast MyHC2X fibre types have been demonstrated in ME/CFS patients.^154^ Similarly, the transcriptome analysis of the ME/CFS skeletal muscle^154^ also showed significant changes in gene transcripts involved in muscle energetic impairment like downregulation of pyruvate dehydrogenase kinase PDK4, a mitochondrial multienzyme complex that catalyzes the first irreversible step of glucose oxidation, and phosphofructo-2-kinase/fructose-2,6-biphosphate PFKFB3 attesting the involvement of mitochondrial metabolism on ME/CFS muscle symptoms. Other data point out metabolic alterations and abnormalities in mitochondrial activity in ME/CFS patients are a deficiency of serum acylcarnitine.^155^ It has been ascertained that glucose levels in ME/CFS plasma are significantly decreased both with respect to healthy and fibromyalgia subjects. As a result, even low-intensity exercise could result in exhaustion for ME/CFS patients. Moreover, less energy disposal could negatively impact excitability or cause metabolic deficiency common in fatigability.

The mitochondrial axis interestingly fits the bioenergetic and metabolic pathways of stress adaptations, since these organelles are integrated with neuroendocrine and metabolic stress mediators, even regulating the psychopathological processes of stress.^156^ Mitochondrial dynamics and immunity are interlinked with each other and this may be a fundamental hub that, along with gut processes, interacts with the circadian rhythm and is possible driver of ME/CFS pathophysiology.^157^

Therefore, the non-linear metabolic dynamics can be altered by external disruptors, thereby determining a disrupted metabolic status. This metabolic model is confirmed by the findings of Naviaux and colleagues, who discriminated this syndrome as a coordinated hypometabolic state resulting from the response to environmental stress, in which several metabolites such as sphingolipids, glycosphingolipids, phospholipids, purines, and flavin adenine dinucleotide are all decreased, as opposite to metabolic syndrome and cell danger response.^158^ The shift from physiological to pathophysiological processes can be nurtured by internal disruptors through interlinked systems, ultimately impairing the possibility of reversing the pathology.

## 4. | CLINICAL FEATURES AND DIAGNOSIS

### 4.1 | The long journey of ME/CFS diagnostic issue

Whether ME and CFS are separate conditions has been a long debate: ME has been referred to as a neuromuscular disease, whereas CFS to a fatigue syndrome, thus leading to consider ME, CFS, and chronic fatigue as three different entities.^159^ The discriminative definition of ME has been proposed to reside on: (1) muscle fatigability/prolonged muscle weakness after trivial exertion, (2) neurological disturbance, especially of cognitive, autonomic, and sensory functions, (3) fluctuation of symptoms, and (4) a prolonged relapsing course. CFS is distinguished from other fatigue diseases by the following diagnostic criteria: unexplained chronic fatigue, perceiving fatigue causes substantial reduction in daily activities, and at least four of the following symptoms: substantial reduction in previous levels of occupational, educational, social, or personal activities; sore throat; tender cervical or axillary lymph nodes; muscle pain and multi-joint pain without joint swelling or redness; headaches of a new type, pattern, or severity; unrefreshing sleep; and PEM lasting more than 24 h.^160^

The debate is still not entirely resolved, although both terms refer to similar symptoms and unknown etiology. The Committee on the Diagnostic Criteria for Myalgic Encephalomyelitis/Chronic Fatigue Syndrome (Institute of Medicine, National Academies, USA) in 2015 proposed to change the name of ME/CFS to systemic exertion intolerance disease (SEID), to better capture the main feature of this disease.^10^ The SEID diagnostic criteria well match recent findings of mild neuro-inflammation and lower levels of metabolites, but produce an overrating of prevalence, thus the complicated journey in the diagnosis of ME/CFS is still continuing.^161^ Difficulty still exists in finding clear disease biomarkers and genetic signatures of the disease. Therefore, reporting the screening laboratory tests and the exclusion cut-off values, along with searching for alternative diagnoses and co-morbidities, are currently suggested for mitigating the impact of misdiagnosis on the reproducibility of ME/CFS findings.^162^ The efforts to obtain generalizable findings force investigators to deal with a plethora of exclusionary conditions, thereby leading to a large consensus on exclusionary illnesses.^163^ As a matter of fact, the differences among case definitions (such as CDC, Holmes, Oxford, Australian, ECD, CCC, PVES, and NICE) and diagnostic methods (such as interviews with/without medical tests, diagnosis by physicians, and medical records) cause huge heterogeneity in the reported prevalence of ME/CFS.^1^ The current algorithm of diagnosis for SEID/ME/CFS consists of (1) functional impairment persisting for more than 6 months accompanied by (2) unexplained fatigue, (3) post-exertional malaise (POM), (4) unrefreshing sleep, (5) cognitive impairment, and/or orthostatic intolerance, and it helps to define a chronic state of multi-systemic disequilibrium similar to post-acute sequelae of viral infections.^164^ Beyond the agreement on exclusionary conditions and the definition of a functional algorithm, there is an urgent need to set specific molecular and biophysical markers that depict the pathophysiological signature of ME/CFS.

### 4.2 | Differential diagnosis

Since fatigue is a prominent feature in a range of medical problems (see Table 1), a detailed clinical characterization, including laboratory and functional tests, is necessary for the differential diagnosis. Indeed, diagnosis is possibly biased by comorbidities if the diagnosed fatigue-based disease does not fully explain symptom’s characteristics.^9^ An imbalance of activity–recovery, persistent negative psychosocial stress, worse pain, impaired abilities to recover from physical or mental exertion, and poorer overall health have been recorded in individuals diagnosed with ME/CFS compared to those with similar symptoms but having no ME/CFS diagnosis.^165^

From a phenomenological point of view, burnout syndromes present similarities with ME/CFS, even though burnout has been historically contextualized within a psychological setting.^166^ Non-resolved fatigue accumulation overlaps with many conditions, including the overtraining syndrome, whose multi-systemic nature is usually analyzed by means of endocrine markers, immune pathways, and electrophysiological evidence.^167^

There are many different flavors of muscle diseases and muscle conditions that develop as co-morbidities. Translational networks of clinical and basic research groups, such as the European Network on ME/CFS (EUROMENE), can support the harmonization of diagnosis and research.^168^ The most known overlapping issue regards ME/CFS and fibromyalgia, which share some symptoms and substantial comorbidity. However, analysis of the skeletal muscle tissue reveals that membrane fluidity was higher in ME/CFS than in fibromyalgia.^54^ Recently, a different lipidomic profile has been highlighted, in particular, plasma ceramide and different metabolites of choline have been found selectively present in ME/CFS plasma and not in fibromyalgia.^169^ Infections have also been under magnifying lens, since infections are known to be a possible cause of long-term impairments, including the occurrence of post-resolution fatigue. A resemblance was found between post-COVID syndrome and ME/CFS, leading to interpretation of ME/CFS as neuroimmune pathogenesis rather than primary mental disorders, with symptoms covering several domains, such as post-exertional exhaustion, immune dysfunction, sleep disturbances, dysfunction of the autonomic nervous system, neurological sensory/motor disorders, and pain syndromes.^170^ The high degree of similarity in the profiles of symptom scores across the so-called post-acute sequelae of SARS-CoV-2 infection (PASC) and ME/CFS can be interpreted as a result of multi-systemic dyshomeostasis. This perspective accounts for fatigue, headache, cognitive dysfunction, post-exertional malaise, orthostatic intolerance, and dyspnea as a consequence of a systemic and hypothalamic immune/inflammatory response, triggered by an external stressor, which results in fluctuating neuroinflammation.^164^

## 5 | PERSPECTIVES: WHERE WE ARE LOOKING AND WHERE WE MIGHT LOOK

### 5.1 | New perspectives on pathophysiological mechanisms

Advancing mechanistic understanding of ME/CFS will help define effective therapeutics since treatments have been mostly palliative so far. Recent efforts toward drug discovery have focused on the identification of multiple targets such as immune dysregulation and mitochondrial dysfunction.^177^ In a review published in 2020, Anderson and Maes focused attention on the interactive role of gut physiology, opioidergic system, miRNA-155, immune cell mitochondria, autonomic nervous system, viral dynamics, leptin, and melatonin, highlighting the importance of circadian rhythms dynamics.^157^

An intriguing perspective lies in the aberrant activation of human endogenous retrovirus (HERVs), that is, dormant viral sequences incorporated in our genome representing about 8% of human genome and found to be differently expressed in ME/CFS patients; this activation can be triggered by external factors, such as infections, and patients may be less capable to re-silence HERVs.^178^ Evidence for post-viral fatigue syndrome, whose criteria were similar criteria to those proposed for CFS, has been known since decades ago, for example, due to a possible persistent enteroviral infection in the muscle,^179^ and muscle fatigue may consequently arise because of the following mitochondrial damage^180^; indeed, the neuromuscular features of ME were suggested to be a complication of subclinical enteroviral infection.^181^ The immune defects and infectious agent hypothesis is still advancing, nurtured by complex interaction between the immune system and viruses/retroviruses of our genome with subtle changes in cytokines, NK cells, and T cells, combined with the induced mitochondrial dysfunction.^178^

A hypothesis lies in autonomic and vascular dysregulation: sympathetic overactivity in the presence of dysfunctional ß2-adrenergic receptors and endothelial dysfunction induces the release of hyperalgesic vasodilators as a compensatory response.^32^ This bioenergetic and vascular hypothesis postulates that metabolic disturbance leads to calcium overload inducing poor energetic situation and endothelial dysfunction in skeletal muscle.^32^

The detection of altered metabolites in the blood such as NAD+ and tryptophan (TRYP) metabolites, stem cells, or extracellular vesicles from ME/CFS patients may be important for novel diagnostic tools.^182,183^ A disturbance in TRYP metabolism, which results in a reduction in NAD+ biosynthesis, was observed in ME/CFS patients, leading to the etiological hypothesis of indolamine-2,3-dioxygenase (IDO) “metabolic trap.”^151^ TRYP is essential in mitochondrial respiration, calcium homeostasis, apoptosis, transcriptional regulation, and immunogenicity.^183^ The abnormal TRYP metabolism observed in ME/CFS patients is an intriguing research topic linked to the etiological hypothesis of immune dysfunction. In fact, about 5% of TRYP is normally metabolized via serotonin and melatonin pathway, and 95% via kynurenine pathway,^184^ with the latter catalyzed by IDO. IDO is expressed in antigen presentation cells, and it could be induced by IFN-y, IFN-α, TNF-α, and LPS.^185^ The depletion of TRYP, mediated by the kynurenine pathway, suppresses the T-cell function by altering the translational program of T-cells and promoting CD4+ T-cell differentiation toward regulatory T-cells.^186^ This results in reprogrammed metabolic pathways (glycolytic, glutaminolytic, and fatty acid oxidative in the mitochondrial matrix).^185^ Recently, it has been proposed that “the metabolic trap hypothesis” for which the genetic mutation of IDO isoforms in ME/CFS patients leads to TRYP over accumulation, blocked kynurenine pathway, and disruption of melatonin and serotonin pathway,^151^ all dysregulations compatible with the clinical presentation and symptomatology of ME/CFS.^182^ All these patterns lead to impaired CNS, gastrointestinal, immune, and energy metabolism functions. In particular, the hypometabolic phenotype of ME/CFS patients could be partially explained by the insufficient synthesis of NAD+ from kynurenine metabolites.^182^ Interestingly, the TRYP trap hypothesis also involves complications in the microbiota. Altering the production of indole metabolites plays a critical role in the integrity of the intestinal mucosal barrier, endotoxin translocation, chronic inflammation, and homeostasis of the immune function, which subsequently affects the gut–brain crosstalk.^187,188^ On the other hand, the abnormal activation of the immune response could lead to an increased IDO activity, resulting in a depletion of TRYP and increased generation of kynurenine and derivatives such as quinolinic acid. This overproduction and consequently central sensitization, along with neuroactive and cytotoxic action,^189^ potentially play a role in the initial fatigue response in ME/CFS.^190^ The accumulation of quinolinic acid could also overactivate poly (ADP-ribose) polymerase (PARP), an enzyme normally involved in repairing DNA damage but depleting NAD+ and ATP production. This leads to mitochondrial impairment, loss of cell energy, and overproduction of ROS and RNS, as observed in ME/CFS.^183^

### 5.2 | New perspectives on topics of interest for diagnostics

Parallel to the effort to study single or a small number of potential biological markers, –omic approaches have been applied to analyze DNA,^95,191^ RNA,^192^ protein,^193–195^ peptide, metabolic products,^158,196–200^ microbiome,^134,189,201–204^ and virome^192,205,206^ in ME/CFS samples, aiming to understand disease pathology and mechanisms, and identify biomarker panels of diagnostic potential (Figure 4). Recently, machine-learning approaches have been adopted to assist analysis of large data sets containing multiple parameters.^193,195,207,208^ In addition to molecular markers, in vitro cell-based assays have been suggested as screening platforms. In one scenario, the cells are used as reporters to detect the existence of metabolic, cytokine, RNA, and other biomolecules in ME/CFS body fluids, based on the rich literature on the altered molecular profiles in these fluids. Morphology, differentiation state, and/or growth patterns of cells are measured to indicate the combined effect of ME/CFS-soluble factors.^169^ In another scenario, dysfunction of ME/CFS cells is measured to reflect pathology associated with the disease. Given the close association of ME/CFS with immune dysfunctions,^194^ immune cells and their biomarkers are popular options for the investigation of ME/CFS pathology, while a number of cell-based assays have been recently evaluated based on other cell types.

#### 5.2.1 | The function of cells cultured in ME/CFS plasma

A few research groups used responses of cells cultured in ME/CFS plasma to reflect the abnormal plasma composition. Peripheral blood mononuclear cells (PBMCs) cultivated with plasma from ME/CFS patients show different electrical properties compared to cells cultured in health control plasma, suggesting cells as reporters to detect ME/CFS-specific plasma factors.^209^ Oxygen consumption of muscle cell lines is also found to be altered upon addition of ME/CFS human plasma. The growth and differentiation of mesenchymal stem cells and induced pluripotent stem cells are further proposed as cell reported given their sensitivity to environmental cues.^169^

#### 5.2.2 | Endothelial dysfunction

Recently, endothelial dysfunction and altered endothelial biomarkers have been found in ME/CFS patients, mimicking observations from patients of post-COVID syndromes (PCS). Characterizing peripheral endothelial function using post-occlusive reactive hyperaemia peripheral arterial tonometry (RH-PAT), Haffke^210^ found endothelial dysfunction and elevated serum endothelin-1 in a subset of post-COVID ME/CFS and PCS patients. Scherbakov et al.^211^ found an association of endothelial dysfunction with the severity of ME/CFS and immune-associated symptoms. In a separate study, Flaskamp^212^ found that a HUVEC cell line exposed to sera from PCS and ME/CFS patients exhibited different secretion profiles and in vitro tube formation, but they did not compare the cell response using healthy donor’s sera.

#### 5.2.3 | Skeletal muscle dysfunction

Skeletal muscle (about 40% of the body mass) can affect the metabolic profile in a syndrome-specific manner. A number of studies have pointed out the dysfunction of skeletal muscles in ME/CFS, as well as changes in the ME/CSF muscle on the molecular,^34^ organelle,^213,214^ cellular,^34,215^ and tissue^216,217^ levels. These include the phenotype of skeletal muscle cells, excitation–contraction cycle, biogenesis of mitochondria,^34^ redox status,^214^ bioenergetic function,^218^ oxidative and nitrosative stress management, metabolic activities,^33^ gene expression, protein synthesis, electrolyte content,^219^ ss2-adrenergic receptor function,^32^ acid handling,^220^ membrane function,^221^ etc. The specific skeletal muscle-derived metabolomic plasmatic profile opens a window of opportunity for biomarker identification. For example, changes in the serum (or plasma) concentrations of certain amino acids have been reported in ME/CFS patients.^222^ In another study, significant decrease in the amounts of several amino acids and metabolites was observed in the urine of ME/CFS patients.^223,224^ To evaluate the cell function, MPC from ME/CFS and control subjects were cultured and tested for AMP kinase (AMPK) activation, metabolic responses to exercise^215^ and substrate utilization, and stimulated contraction^218^ in vitro. Impaired AMPK activation, increased glucose uptake and IL6 release, and increased myogenin expression were noted in ME/CFS cells, suggesting genetic/epigenetic contributions. The same in vitro system was also used to test pharmacological activation of AMPK in a later study.^225^ In addition, human MPCs could be fundamental in revealing a specific secretome signature. For example, ceramide, an essential component in the formation of exosomes and extracellular microvesicles, has been shown to elevate in ME/CFS.^200^

Following the eagerness to find new pathways of interest, attention has to be paid to the metabolism of tetrahydrobiopterin by the guanosine triphosphate cyclohydrolase I (GTPCH) enzyme. This enzyme is strictly dependent on the production of intracellular GTP, which is conspicuously and selectively synthetized by means of succinyl-CoA synthetase on mitochondrial succinyl-CoA transformation. This may spur new investigations linked to axis mitochondria/metabolism/fatigue.^226^

#### 5.2.4 | Altered cell response to stress

Hypoosmotic stressors have been shown to induce different stresses in peripheral blood mononuclear cells from ME/CFS patients compared to those from health controls. The differences have been detected using a nanoneedle bioarray.^209^ In our group, model muscle cell lines chemically treated to present elevated oxidative stress^227^ or intracellular calcium levels^228^ have also been shown to present distinct electrical signatures compared to untreated cells. Since elevated oxidative stress and alteration in calcium signalling are typical characteristics of ME/CFS skeletal muscle tissues, the electrical characteristics of muscle cells have been proposed as a signature to define ME/CFS muscle dysfunction, while this idea has yet to be tested using clinical samples.

#### 5.2.5 | Red blood cell deformability

Based on the evidence of oxidative damage in haematological systems in ME/CFS, red blood cell (RBC) deformability has been measured using a microfluidic platform and high-speed microscopy.^229^ RBCs from ME/CFS patients have been found to be significantly stiffer than those from healthy controls, which is suggested as a first-pass diagnostic test.

#### 5.2.6 | New engineering approaches

Besides the effort to expand the type of biomarkers, advances have also been made to apply new techniques to detect these biomarkers, especially techniques that are simple to practice and cost-effective. Raman spectroscopy has been implemented due to its capability to offer label-free biochemical information. Gonzalez-Cebrian et al. used Raman micro-spectroscopic analysis to identify fingerprints from ME/CFS extracellular vesicles. The carotenoid peaks are highlighted as the main difference between severe ME/CFS patients and healthy controls. These peaks are proposed to reflect erythrocyte deficiencies in ME/CFS patients.^230^ Xu et al. analyzed single peripheral blood mononuclear cells using Raman microspectroscopy and identified Raman bands associated with phenylalanine are significantly higher in ME/CFS patients than in healthy controls. The difference is related to mitochondrial/energetic dysfunctions.^231^

## 6 | FUTURE PERSPECTIVES AND CONCLUSION

In conclusion, ß2-adrenergic receptor dysfunction which triggers cytosolic calcium overload, similarity physiological compromise at the musculoskeletal level in patients and elderly, and dysfunctional membranes all raise skeletal muscle as a major player of ME/CFS syndrome. Current diagnosis of ME/CFS depends on symptom-specific case criteria following the exclusion of any other explanatory diagnosis, which suffers from the highly heterogenous symptoms. Potential ME/CFS biomarkers studied so far differed in efficiency, quality, and translatability in their diagnostic power. Heterogenous results are often seem among the patients.^232^ Moreover, inconsistent and irreproducible findings are found across different studies,^195,233^ some studies lack strict case definitions to define pathophysiology,^234^ many studies require validation of findings with larger sample sizes and age- and gender-matched controls.^98,195^ Linking the biomarkers with the pathogenesis and etiology of ME/CFS is another challenge and is critical for understanding their diagnostic power and treatment options. Therefore, a deeper investigation into muscle-specific biomarkers could help decipher the yet-undefined ME/CFS on physiological and clinical bases.

Longitudinal studies should be performed to characterize how the biomarkers evolve and develop with the disease progression and severity. The heterogeneity across many of the studies highlights the need for multi-disciplinary research and uniform protocols for ME/CFS research.^235^ In addition, databanks are needed for comprehensive understanding of the disease.^236^ Interesting perspectives lie on the immune defects and infectious agent hypothesis, bioenergetic, and vascular hypothesis based on ß2-adrenergic receptor, and the metabolic trap hypothesis based on indolamine-2,3-dioxygenase. Despite the major role of skeletal muscle in ME/CFS, this tissue is still underrepresented in diagnostic algorithms; therefore, investigating ME/CFS skeletal muscle characteristics, particularly on epigenetic and bioelectrical bases, is promising for identifying new valid diagnostic markers. New evidence from skeletal muscle research could also resolve the long-standing debate about the possible discrimination among the entities of ME, CFS,^237^ SEID, and other disorders in which fatigue plays a central role.

## Supplementary Material

Supplementary Table

Additional supporting information can be found online in the Supporting Information section at the end of this article.

## Figures and Tables

**FIGURE 1:**
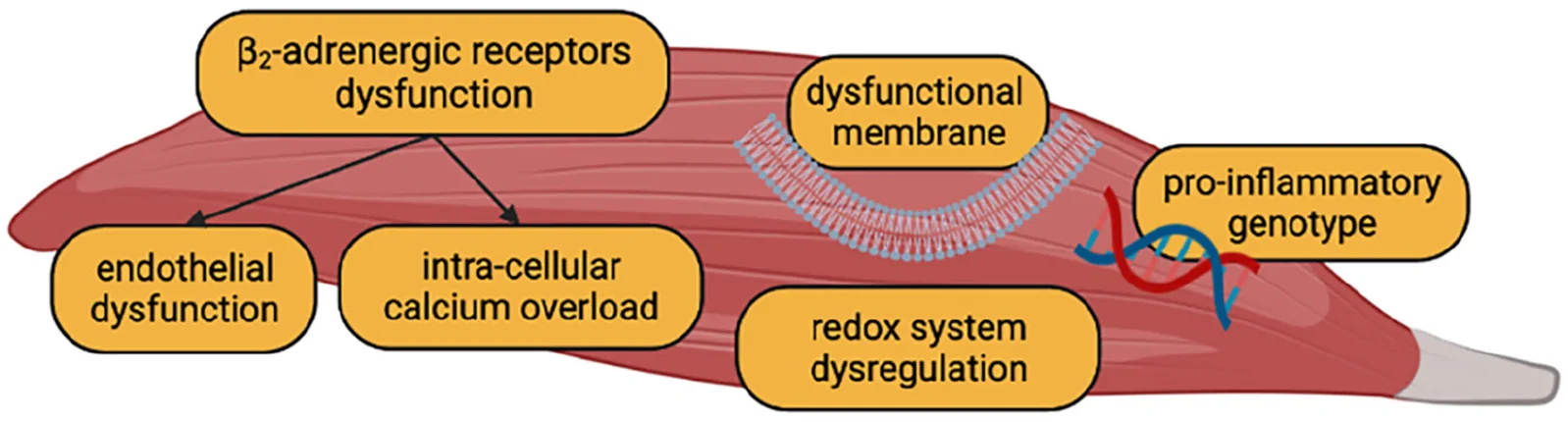
Putative mechanism of ME/CFS targeted on skeletal muscles. Credit: BioRender.

**FIGURE 2:**
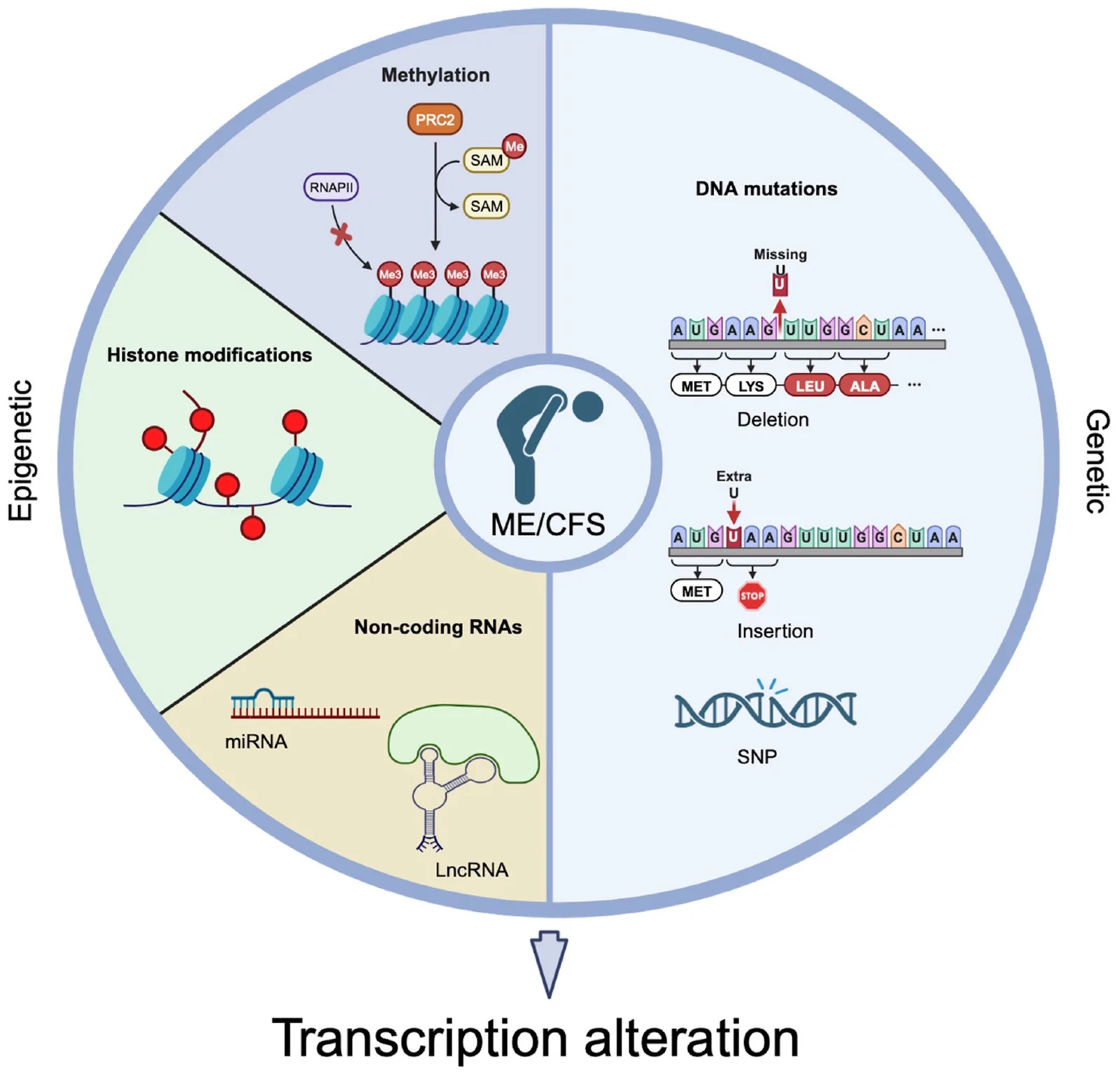
Representation of the two opposite molecular mechanisms (epigenetic and genetic) that cause transcriptional alterations. Both epigenetic and genetic alterations were associated with the development of ME/CFS. The figure represents DNA methylation, histone modifications, and miRNA-target interaction and the interaction between a protein-regulating DNA accessibility with a long non-coding RNA (lncRNA) within the epigenetic mechanisms. As genetic mechanisms show DNA deletions, insertions, and single nucleotide polymorphisms (SNPs). Credit: BioRender.

**FIGURE 3:**
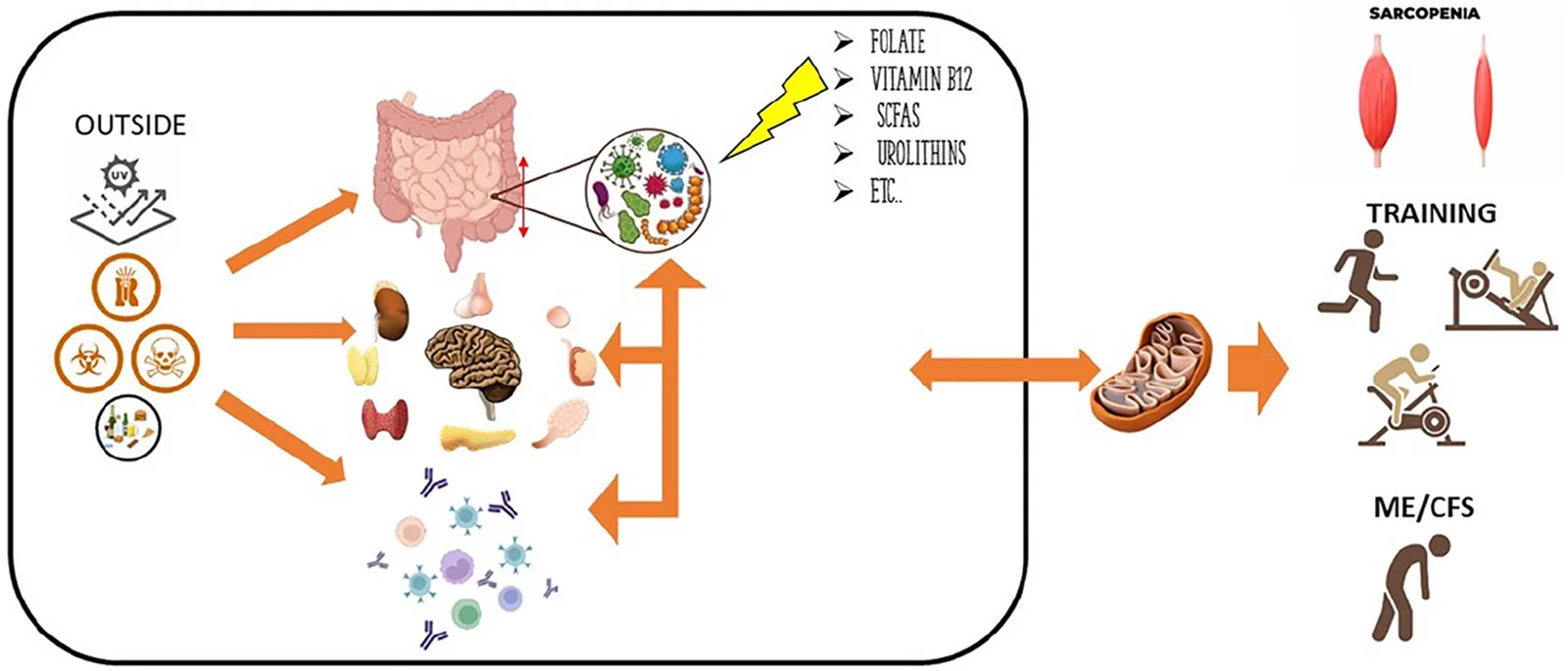
Exposome and internal pathways interact to determine mitochondrial status, thereby resulting in either health or diseases. Credit: BioRender.

**FIGURE 4:**
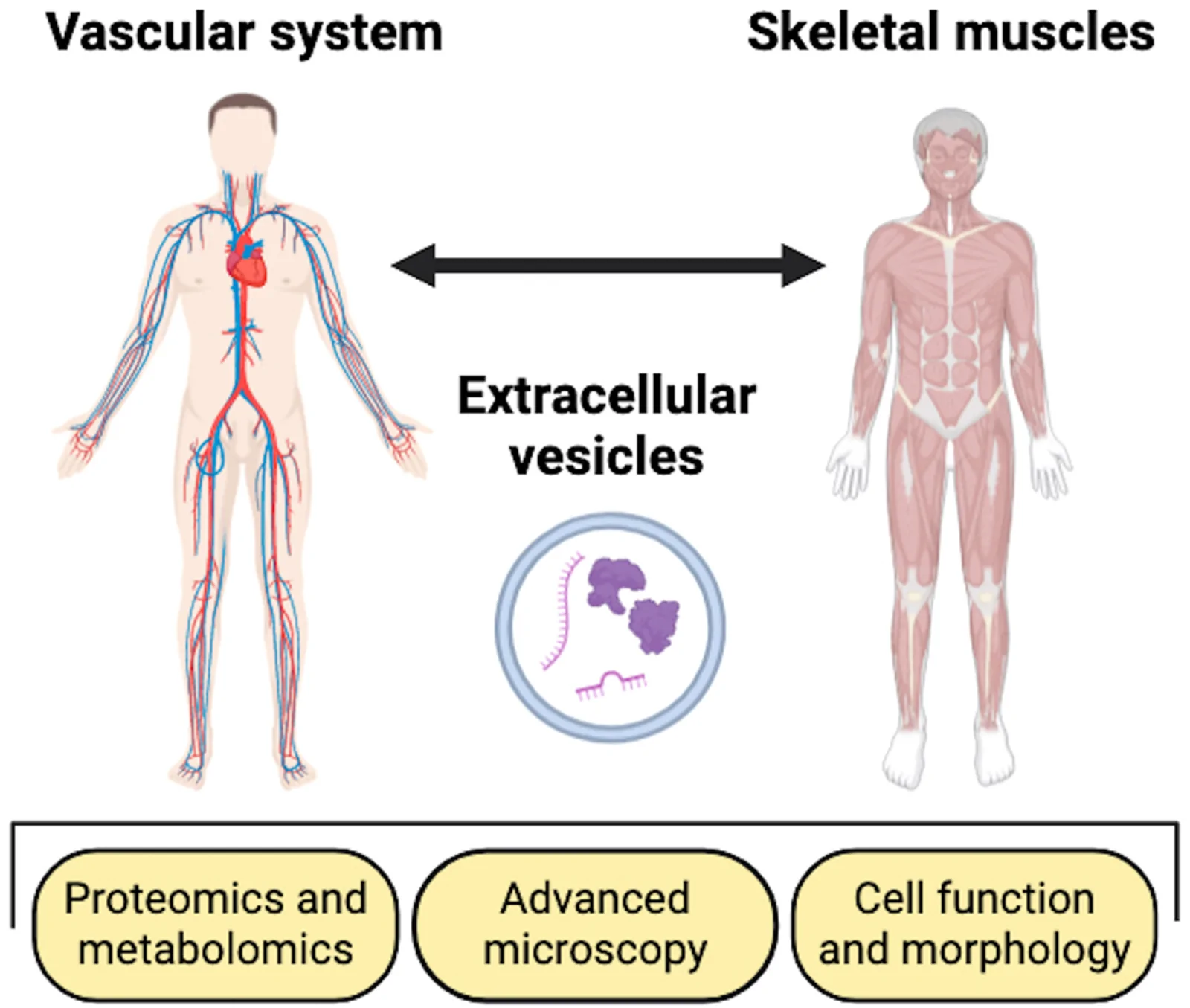
Biological entities of interest and analytical methods for advancing ME/CFS diagnostics. Credit: BioRender.

**TABLE 1:** Differential readouts and management of some diseases characterized by fatigue.

	Diagnosis	Treatment	Ref.
Fibromyalgia	• Pain (present or recurrent for longer than 3 months) • Hypersensitivities	• Non-pharmacological approach (strong recommendation for physical exercise) as first-line therapy	[171,172]
McArdle disease (glycogen storage disease type V)	• Forearm ischaemic exercise test (lack of elevation of blood lactate levels) • Excess of glycogen • Deficit of myophosphorylase activity in the muscle biopsy • Genetics analysis of PYGM in white blood cells	• Non-intense aerobic exercise • Ketogenic and protein-rich diets only when the patients had already suffered an episode of rhabdomyolysis	[173]
Multiple sclerosis	• EDSS • MRI to detect central nervous system demyelination • Oligoclonal banding	• Interferon β-1b, interferon β-1a intramuscularly, interferon β-1a subcutaneously, and glatiramer acetate to reduce number of lesions and new lesions	[174]
Inflammatory myopathies	• 6MWDT • FI-3 scale • Alterations of muscle architecture, with fat infiltration, fibrotic replacement, and muscle atrophy	• Supervised physical activities • Avoiding excessive loading	[175]
Rheumatoid arthritis	• Elevated levels of CRP and ESR in serum • Detection of RA-specific autoantibodies • Ultrasound and MRI for detecting synovial hypertrophy and bone erosion	• NSAIDs • Immunosuppressive glucocorticoids • DMARDs	[176]

*Note*: Psychological assistance, in spite of its relevance, has not been included since it goes beyond the aims of this review.

Abbreviations: CRP, C-reactive protein; DMARDs, disease-modifying anti-rheumatic drug; EDSS, expanded disability status scale; ESR, erythrocyte sedimentation rate; FI, functional index; NSAIDs, non-steroidal anti-inflammatory drugs; PYGM, glycogen phosphorylase, muscle associated; RA, rheumatoid arthritis; 6MWDT, 6 minutes walking distance test.

## Data Availability

N/A.
